# Diagnostic efficacy of metagenomic next generation sequencing in bronchoalveolar lavage fluid for proven invasive pulmonary aspergillosis

**DOI:** 10.3389/fcimb.2023.1223576

**Published:** 2023-08-24

**Authors:** Hongxia Jia, Hongping Liu, Meng Tu, Yan Wang, Xinjuan Wang, Jing Li, Guojun Zhang

**Affiliations:** Department of Pulmonary and Critical Care Medicine, the First Affiliated Hospital of Zhengzhou University, Zhengzhou, China

**Keywords:** metagenomic next generation sequencing, BALF, invasive pulmonary aspergillosis, ROC curve, diagnostic efficacy

## Abstract

**Objective:**

To assess the diagnostic efficacy of metagenomic next generation sequencing (mNGS) for proven invasive pulmonary aspergillosis (IPA).

**Methods:**

A total of 190 patients including 53 patients who had been diagnosed with proven IPA were retrospectively analyzed. Using the pathological results of tissue biopsy specimens as gold standard, we ploted the receiver operating characteristic (ROC) curve to determine the optimal cut-off value of mNGS species-specific read number (SSRN) of *Aspergillus* in bronchoalveolar lavage fluid (BALF)for IPA. Furthermore, we evaluated optimal cut-off value of mNGS SSRN in different populations.

**Results:**

The optimal cut-off value of *Aspergillus* mNGS SSRN in BALF for IPA diagnosis was 2.5 for the whole suspected IPA population, and 1 and 4.5 for immunocompromised and diabetic patients, respectively. The accuracy of mNGS was 80.5%, 73.7% and 85.3% for the whole population, immunocompromised and diabetic patients, respectively.

**Conclusions:**

The mNGS in BALF has a high diagnostic efficacy for proven IPA, superioring to *Aspergillus* culture in sputum and BALF and GM test in blood and BALF. However, the cut-off value of SSRN should be adjusted when in different population.

## 1 Introduction

The genus Aspergillus, consisting of 350 species, can be divided into six subgenera, of which Aspergillus is the most common subgenus. Aspergillus is a saprophytic fungus found in a variety of habitats, such as soil, compost, and dust (Houbraken et al., 2020). Aspergillus species are the most common cause of pulmonary mycosis with a broad spectrum of clinical presentations. The most common causative subgenus is the A. fumigatus species complex, followed by Aspergillus flavus, Aspergillus terreus and Aspergillus niger (Lass-Florl et al., 2021). Because Aspergillus is widespread in the environment, Aspergillus conidia easily enter the body through the respiratory tract, and infection can occur locally or spread to adjacent or distant sites by aspiration of the spores. Pulmonary aspergillosis includes allergic bronchopulmonary aspergillosis (ABPA), chronic pulmonary aspergillosis (CPA), and invasive pulmonary aspergillosis (IPA). ​IPA occurs primarily in immunocompromised individuals, but also in some immunocompetent individuals. ​It is a major cause of morbidity and mortality in patients, especially in patients with neutropenia following glucocorticoid therapy, immunosuppressive therapy, bone marrow transplantation, or chemotherapy.​ Diagnosis of IPA is graded according to probability (possible, probable, or proven), due to the lack of typical clinical signs and the variable sensitivity and specificity of radiological and mycological tests. According to The European Organization for Research and Treatment of Cancer and Mycoses Study Group Education and Research Consortium (EORTC-MSGERC), probable IPA can be diagnosed according to host, clinical and mycological criteria, and only in immunocompromised patients. Possible IFD can be considered if the cases only meet the host and clinical criteria, although the definition of possible IFD is still controversial. A diagnosis of proven IPA can be made by the presence of hyphal or melanized yeast-like forms in histopathology accompanied by evidence of associated tissue damage. The definition of proven IPA applies to any patient, regardless of the patient's immune status (Donnelly et al., 2020). 

Fungal detection methods for the diagnosis of IPA include fungal culture, specific polymerase chain reaction (PCR) or galactomannan antigen(GM)test. Despite all these mycological trials, diagnosis of IPA is challenging. The GM test is a relatively specific biomarker for IPA diagnosis, which can be detected in serum and BALF. Data on the sensitivity and specificity of this test vary. Relevant studies showed that the sensitivity and specificity of this test in serum was 58%-92% and 85%-95%, respectively. The sensitivity and specificity of this test in BALF was 75%-86% and 93%-100%, respectively (Zhou et al., 2017). But all of those studies were performed in specific cohorts, such as immunosuppressed population and typically in probable IPA, not for proven IPA. The positive rates of sputum culture and BALF culture were low (Lamoth and Calandra, 2022). PCR has been used to assist in the diagnosis of invasive aspergillosis for more than 20 years and has been included in EORTC/MSG definitions of IPA since 2020. However, the performance of PCR was similar to GM test (White et al., 2015). Microscopy and culture in sputum or BALF are routine microbiological methods for identifying fungi, but culture is time-consuming and both of them are relatively insensitive. Evidence of fungal infection in histopathology is the gold standard for the diagnosis of IPA. However, lung biopsy is an invasive test that is not suitable for all patients.

Metagenomic next-generation sequencing (mNGS) produces millions to billions of readings per instrument run by directly sequencing several individual DNA molecules in parallel, regardless of their composition. Adenine, guanine, thymine, and cytosine are the four continuous bases that make up reads, the fundamental product of DNA sequencing (Simner et al., 2018). Following the completion of the sequencing, offline data were automatically identified using software for analyzing pathogenic microorganism sequencing data, data quality control was carried out on the raw data, human hosts were eliminated, and comparisons with a self-built pathogen database were made for species identification (Chiu and Miller, 2019).​ The sequencing data were analyzed for species specific read number (SSRN) and genome coverage (%), and the Aspergillus depth with SSRN≥1 was considered positive. The data interpretation method has been described in detail elsewhere (Xing et al., 2020).

At present, mNGS technology is increasingly used in the clinical diagnosis of infectious diseases, especially when the pathogens of infectious diseases are not available or the anti-infective treatment is not effective. Many studies have shown the diagnostic value of mNGS in virus infections, but little research has been done on pulmonary fungal infections, especially *Aspergillus* infections. Interpretation of mNGS results is also difficult due to the frequent presence of oral microbiota and colonized micro-organismsin the respiratory specimens (S. et al., 2017; L. et al., 2018; Miao et al., 2018). To the best of our knowledge, the diagnostic efficacy of mNGS for pulmonary aspergillosis has not been investigated using histopathological findings as the gold standard. Hence, we conducted this study to evaluate the diagnostic efficacy and cut-off value of mNGS for proven invasive pulmonary Aspergillosis and the differences among different populations.

## 2 Methods

### 2.1 Study design

Inclusion, exclusion and diagnostic criteria

**Figure 1:**
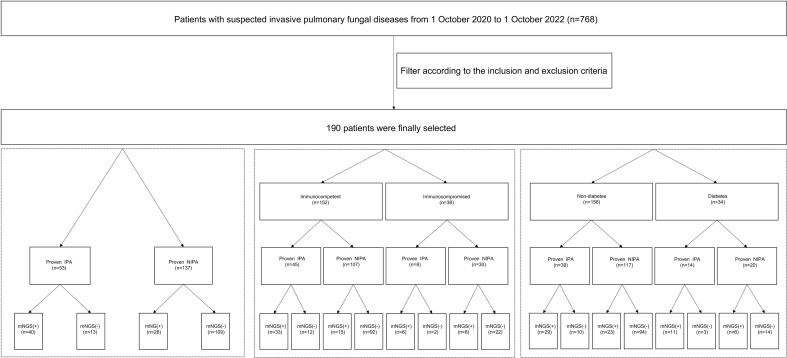
Flow chart of the research design. A total of 768 patients with suspected invasive pulmonary fungal diseases were screened out. Finally190 patients were finally selected by inclusion and exclusion criteria. We take the mNGS as positive when the reads number of the strict mapping reads number genus was no less than 1. IPA, invasive pulmonary aspergillosis; non-IPA-PD, non-invasive pulmonary aspergillosis-pulmonary disease; mNGS, metagenomic next generation sequencing; BALF, bronchoalveolar lavage fluid.

This research was a retrospective study. First, the electronic medical records system was searched to collect 768 patients with suspected invasive pulmonary fungal disease who were hospitalized in the First Affiliated Hospital of Zhengzhou University from October 1, 2020 to October 1, 2022. According to the inclusion and exclusion criteria, 190 patients were finally enrolled in the study. Inclusion criteria:1. Age≥ 18 years. 2. Patients who had taken both lung biopsy and BALF for mNGS and the detection time of lung biopsy and BALF mNGS wasn’t more than one week. 3. The lung biopsy and BALF mNGS were performed before antifungal administration. Exclusion criteria: the type of fungus cannot be distinguished by lung histopathology. Finally, 190 patients were finally selected (**Figure 1**). According to EORTC/MSG (Donnelly et al., 2020), the positive of septate hyphae in histopathological staining and in the culture in sterile site are the criteria for proven case. ​Proven IPA diagnosis criteria in our study: pathological results of lung tissue specimens obtained from needle aspiration or surgical biopsy revealed septate hyphae, confirmed as *Aspergillus* by two independent pathologists based on *Aspergillus* morphological features, and accompanied by evidence of associated tissue damage.​ ​The risk factors for immunocompromised patients are as follows: administration of steroids for more than 3 months or at doses greater than 0.5 mg/kg/d or other immunosuppressants, transplantation of a solid organ, chemotherapy for solid tumors within 5 years, diagnosis of hematological malignancy, and primary immunodeficiency. The diagnosis of diabetes was made according to the WHO diabetes diagnosis standard in 1999. We defined the mNGS result as positive when the species-specific read number (SSRN) of *Aspergillus* was greater than or equal to 1. We also performed GM test and fungal culture in BALF for auxiliary diagnosis. The diagnostic efficacy of mNGS in BALF were analyzed in all patients, and then in immunocompromised population and diabetes population (**Figure 1**). All clinical procedures were performed according to the Declaration of Helsinki’s principles. The First Affiliated Hospital of Zhengzhou University’s institutional ethics committee gave its approval for this study(2023-KY-0365).

### 2.2 Methods of obtaining bronchial lavage solution

The bronchoalveolar lavage was performed according to standard procedures. After clearing the airway, the end of the bronchoscope was embedded in the opening of the bronchial or sub-segment. The total amount of 37 degrees Celsius saline was rapidly injected through the operating aperture, and the total amount of 40ml was injected twice. After each injection, BALF was obtained by appropriate negative pressure suction, and the total recovery rate was ≥30%. The second collected BALF was collected for mNGS.

### 2.3 mNGS procedures for specimens

The collected BALF was sent to the laboratory for mNGS testing within 2 hours and the procedures were performed according to the routine operation of mNGS in our gene hospital Laboratory, which includes sample pretreatment, nucleic acid extraction, library preparation, sequencing and data analysis. In simple terms, first, the experimental instruments were cleaned, disinfected, and treated with nucleic acid scavenger. A total of 800 μL of alveolar lavage solution was taken (the sticky sample was liquefied with enzyme liquefaction solution), transfer the samples to a 2 mL Lysing Matrix E tube (manufactured by MP Company) with 1 g of 0.5 mm glass bead. Utilize the Jingxin JXFSTPRP-4D wall-breaking instrument (Shanghai Jingxin Industrial Development Co., Ltd.) to initiate the treatment program at 18 m/s for a duration of 420 seconds, aiming to physically break the sample cell walls. Then the Humoral microbial DNA Kit (PMD101, Nanjing Practice Medicine Diagnostics Co., Ltd) was used to extracted DNA. After that, the Qubit^®^ 1×dsDNA HS Assay Kit and Qubit 4.0 fluorometer were used to measure the nucleic acid concentration. According to the standard process quality control standard, the quality control concentration of DNA in BALF was 0.2 to 100 ng/μL. Qubit concentration, the concentration of DNA extracted from all samples in our research met the quality control standards. DNA fragments were enzymatically sheared into 200-300 bp lengths using the MGI VAHTS Universal Plus DNA Library Prep Kit (NDM617, Vazyme, China). Following fragment size optimization, end-repair was performed. Subsequently, DNA libraries were constructed through adapter ligation and PCR amplification. Quality control of the DNA libraries was conducted using the Qubit 2.0 fluorometer (Thermo Fisher Scientific, USA) to ensure library concentration exceeded 1 ng/μl.

Eligible double-stranded DNA libraries were converted into single-stranded circular DNA libraries by DNA denaturation and cyclization. Then the DNA nanospheres (DNB) were prepared according to available formulations. Finally, each DNB was loaded into a lane for sequencing on the BGISEQ-200 platform (BGI, Shenzhen, China) (Drmanac et al., 2010). For quality control, raw reads were filtered by fastp v.2.20.0 with default parameters (Chen et al., 2018). After adapter trimming and filtering low-quality sequences, the clean reads that mapped to human reference assembly GRCh38 with bowtie2 v2.5.1 (Langmead and Salzberg, 2012) were removed. Here, we designed a curated pathogen reference databases for selecting representative genomes from the NCBI GenBank genome database (Shi et al., 2020). For each species, only one typical strain sequences were selected from the assembly_summary.txt file, when annotated with the refseq_category= ‘reference genome’ or ‘representative genome’ and the assembly_level= ‘Complete genome’, ‘Chromosome’ or ‘scaffold’ (Jing et al., 2021). Currently, our curated database contains 12,895 bacterials, 11,120 viral taxa, 1,582 fungal taxa, 312 parasites, 184 mycoplasma chlamydia taxa, and 177 mycobacteriums. The remaining reads were aligned to the pathogen reference databases with parameters ‘-Y -h 1000’ by BWA v0.7.17 (Li and Durbin, 2010). Then, we implemented the lowest common ancestor (LCA) annotation strategy to define taxon-specific reads by an in-house python pipline, as previously reported (Jing et al., 2021). Finally, the species-specific read number (SSRN) of *Aspergillus* was obtained.

### 2.4 Statistical analysis of the data

The chi-square test was used for categorical variables. The Kolmogorov-Smirnov test was used to check the normality of the data distribution. A t-test was applied to the normally distributed variables. The Wilcoxon signed rank test was used for non-normally distributed variables. The diagnostic performance of mNGS was measured by area under the curve (AUC) of receiver operating characteristic (ROC). Sensitivity, specificity, positive predictive value, negative predictive value, positive likelihood ratio, negative likelihood ratio, accuracy, and the AUC were calculated for comparisons among different subpopulations. Statistical data were processed and analyzed using SPSS 26.0 (IBM Corp.). A P value of <.05 (2-sided) was considered statistically significant.

## 3 Results

### 3.1 General characteristics

**Figure 2:**
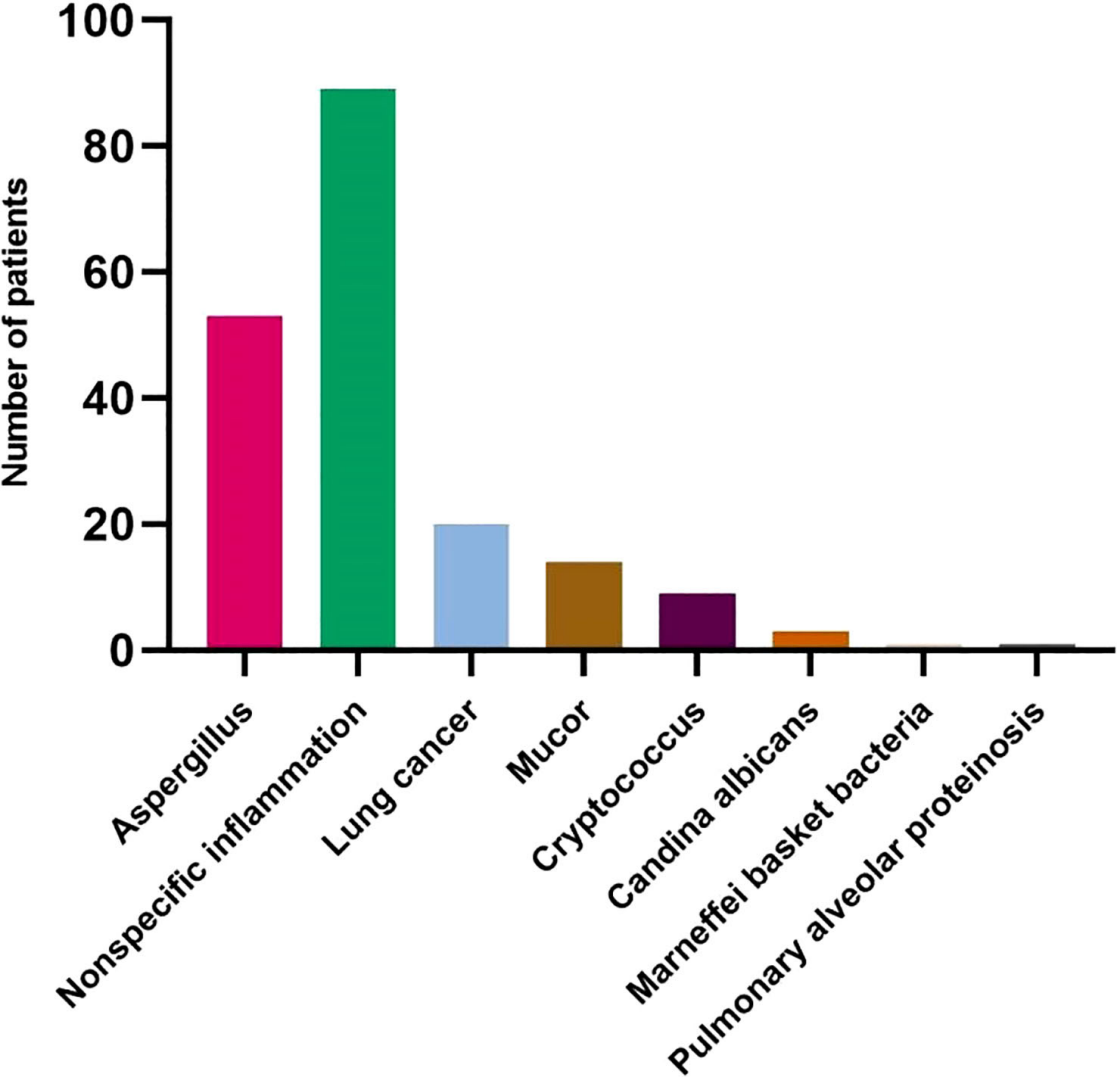
The distribution of lung histopathological in 190 patients.

A total of 190 patients were enrolled, including 53 patients with proven IPA and 137 patients with proven non-invasive pulmonary aspergillosis-pulmonary disease (non-IPA-PD). Among the specimens with histologically proven non-IPA-PD, 89 were nonspecific inflammation, 20 were lung cancer, 14 were Mucor, 9 were Cryptococcus, 3 were Candida Albicans, 1 was Marneffei basket bacteria and 1 was pulmonary alveolar proteinosis (**Figure 2**). The general characteristics of the two groups of patients are shown in **Table 1**. The variables of hemoptysis, CRP and Lymphocytes had significant differences between the proven IPA and proven non-IPA-PD patients. The mNGS had good differential diagnosis value between proven IPA and proven non-IPA-PD (*P* < 0.05), while the variables of GM test in serum, GM test in BALF, sputum-culture and BALF-culture had no significant difference between two groups (*P* > 0.05; **Table 1**).

**Table 1:** The general characteristics of patients.

Variable	Proven-IPA (n=53)	Proven non-IPA-PD (n=137)	P
Age (y), m ± sd	49.28 ± 16.64	53.87 ± 13.32	0.076
Male,n (%)	28 (52.8)	87 (63.5)	0.177
Cough,n (%)	43 (81.1)	106 (77.4)	0.572
Expectoration,n (%)	34 (64.2)	81 (59.1)	0.525
Fever,n (%)	25 (47.2)	61 (44.5)	0.743
Tightness,n (%)	19 (35.8)	61 (44.5)	0.277
Hemoptysis,n (%)	13 (24.5)	17 (12.4)	0.04
Diabetes,n (%)	14 (26.4)	20 (14.6)	0.057
Immunocompromised,n (%)	8 (15.1)	30 (21.9)	0.293
Smoking,n (%)	16 (30.2)	44 (32.1)	0.798
Neutrophils*10^9/L,median (IQR)	4.19 (2.57,10.74)	4.68 (3.28,7.79)	0.533
Lymphocytes*10^9/L,median (IQR)	1.10 (0.73,1.68)	1.48 (0.95,1.92)	0.017
CRP (mg/L), median (IQR)	52.62 (6.20,111.60)	17.24 (3.10,61.19)	0.019
PCT (ng/mL), median (IQR)	0.06 (11.00,0.01)	0.07 (18.05,0.01)	0.686
GM test in serum,positive, n (%) ^a^	3 (7.0)	7 (6.0)	0.828
GM test in BALF, n (%) ^b^	6 (10.7)	6 (20.7)	0.21
Sputum-culture (positive), n (%) ^c^	1 (2.0)	8 (6.4)	0.225
BALF-culture (positive), n (%) ^d^	7 (14.0)	24 (20.0)	0.356
mNGS,positive,n (%)	39 (73.6)	23 (16.8)	0.000

IPA, invasive pulmonary aspergillosis; non-IPA-PD, non-invasive pulmonary aspergillosis-pulmonary disease; m, mean; sd, standard deviation; IQR, Interquartile range; CRP, C-reactive protein; PCT, procalcitonin; GM, glactomannan; mNGS, metagenomic next generation sequencing; BALF, bronchoalveolar lavage fluid. ^a^Data available for 159 patients; ^b^Data available for 85 patients; ^c^Data available for 176 patients; ^d^Data available for 170 patients.

### 3.2 Diagnostic efficacy of mNGS in BALF for all patients

**Figure 3:**
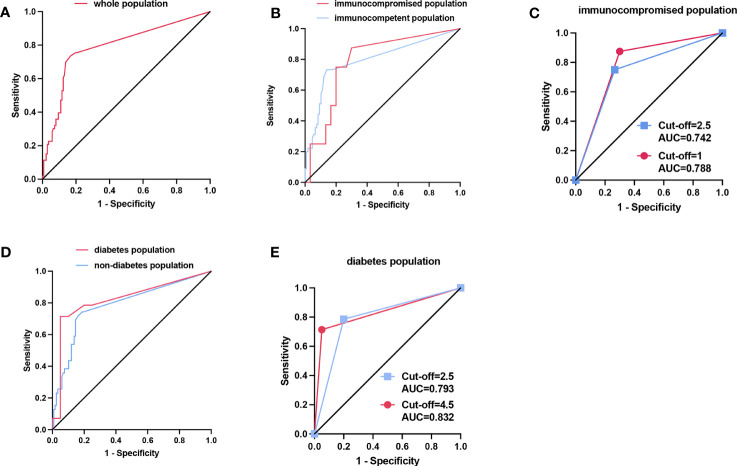
**(A)** The ROC curve of the whole populationwith cut-off value of the SSRN of mNGS was 2.5. **(B)** The ROC curve of the population with different immune states. The optimal cut-off value was 2.5 in immunocompetent population while the optimal cut-off value was 1 in immunocompromised population when the Youden index is at its maximum. **(C)** In the immunocompromised population, the AUC was 0.7417 when the cut-off value was 2.5 (blue line), while the AUC was 0.7875 when the cut-off value was 1 adjusted according to the maximum Youden index (red line). **(D)** The ROC curve of the population with and without diabetes. The optimal cut-off value was 2.5 in the population with no diabetes while the optimal cut-off value was 4.5 in the population with diabetes when the Youden index is at its maximum. **(E)** In the diabetes population, the AUC was 0.7929 when the cut-off value was 2.5 (blue line), while the AUC was 0.8321 when the cut-off value was 4.5 adjusted according to the maximum Youden index (red line).

ROC curve was plotted according to the *Aspergillus* SSRN of mNGS in BALF for all patients enrolled (**Figure 3A**). When the youden index was at its maximum point, the cut-off value of mNGS SSRN was 2.5, and the mNGS in BALF had a high diagnostic efficiency with AUC of 0.790(95%CI: 0.712,0.867), sensitivity of 73.6%, specificity of 83.2%, PPV of 62.9%, NPV of 89.1%, PLR of 2.788, NLR of 0.317, and diagnostic accuracy of 80.5% (**Table 2**). ​A cut-off value of 1 would generate the ultimate sensitivity with 75.5% while a cut-off value of 372238 would generate the ultimate specificity with 100%.

**Table 2:** Diagnostic efficacy of mNGS in BALF in different populations.

	AUC (95%CI)	Sensitivity (%)	Specificity (%)	PPV (%)	NPV (%)	PLR	NLR	Accuracy (%)
All patients (cut-off=2.5)	0.790 (0.712-0.867)	73.6	83.2	62.9	89.1	2.788	0.317	80.5
Immunocompromised (cut-off=2.5)	0.787 (0.617,0.958)	75.0	73.3	42.9	91.7	3.000	0.341	73.7
Immunocompromised (cut-off=1,adjusted by Youden index)	0.787 (0.617,0.958)	87.5	70.0	43.8	95.5	7.000	0.179	73.7
Immunocompetent (cut-off=2.5)	0.795 (0.710,0.881)	73.3	86.0	68.8	88.5	2.745	0.310	82.2
Diabetes (cut-off=2.5)	0.823 (0.663,0.983)	78.6	80.0	73.3	84.2	3.673	0.268	79.4
Diabetes (cut-off=4.5,adjusted by Youden index )	0.823 (0.663,0.983)	71.4	95.0	90.9	82.6	2.497	0.301	85.3
Non-diabetes (cut-off=2.5)	0.786 (0.696,0.876)	71.8	83.8	59.6	89.9	2.546	0.337	80.8

AUC, area under curve; PPV, positive predictive value; NPV, negative predictive value; PLR, positive likelihood ratio; NLR, negative likelihood ratio.

### 3.3 Diagnostic efficacy of mNGS in BALF for different populations

The mNGS in BALF also had a high diagnostic efficiency when applied to different populations. The population was divided into immunocompromised and immunocompetent groups based on immune status, and into diabetic and non-diabetic groups based on the presence or absence of diabetes. Then we also plotted the ROC curve (**Figures 3B, D**). The AUC was 0.787(95% CI: 0.617,0.958) in the immunocompromised group and was 0.795 (95% CI: 0.710,0.881) in the immunocompetent group. The AUC was 0.823(0.663,0.983) in the diabetes group while the AUC was 0.786(95% CI: 0.696,0.876) in the non-diabetes group. When the cut-off value was taken as 2.5 in all different groups, the sensitivity, specificity, and accuracy of immunocompromised group were 75.0%, 73.3% and 73.7%, respectively, and of immunocompetent group were 73.3%, 86.0% and 82.2%, respectively; the sensitivity, specificity, accuracy of diabetes group was 78.6%, 80.0% and 79.4%, respectively, and of non-diabetes was 71.8%, 83.8% and 80.8%, respectively.

Interestingly, when cut-off value was adjusted according to the Youden Index at its maximum point, the immunocompetent group and the non-diabetes group had the same cut-off value, consistent with the whole patients. However, the cut-off values were different according to the Youden Index at its maximum point in immunocompromised group and the diabetes group. In the immunocompromised group, when the cut-off value was adjusted as 1 according to the Youden, the AUC, sensitivity, specificity, and accuracy were 0.788, 87.5%, 70.0% and 73.7%, respectively. Compared with the cut-off value of 2.5, the accuracy had no shift, but the AUC and sensitivity had a significant increase, and the specificity had a slight decrease (**Table 2**; **Figure 3C**). For the diabetes group, when the cut-off value was 4.5 adjusted by the Youden Index, the sensitivity, specificity, and accuracy were 71.4%, 95.0% and 85.3%, respectively. Compared with the cut-off value of 2.5, the AUC, accuracy and the specificity had a remarkable increase with a mild decrease in sensitivity (**Table 2**; **Figure 3E**).

## 4 Discussion

Fungal infections, including IPA, have always been a major threat to human health (Fisher et al., 2018). Timely and accurate diagnosis of IPA remains a great challenge, which facilitates precision treatment (Thompson and Young, 2021). With the aging of the population, the application of broad-spectrum antibiotics, antineoplastic drugs, immunosuppressants, as well as the widespread use of invasive examinations and organ transplantation, the incidence of IPA has increased (Sindhu et al., 2019; Cabrera et al., 2022; Hlophe et al., 2022). The diagnosis of IPA includes three levels: proven, probable, and possible. The proven diagnosis depends on histopathological biopsy, microscopic analysis of sterile material, culture of sterile material, blood culture, tissue nucleic acid diagnosis (Donnelly et al., 2020). Due to absence of typical clinical presentation, the low vigilance of IPA in clinical practice, and the difficulty in obtaining qualified specimens, IPA is easily underdiagnosed and misdiagnosed (Davies et al., 2022; Ekeng et al., 2022), resulting in patients missing treatment opportunities and even death (Lowes et al., 2017). Therefore, it is urgent that novel detection methods are needed to meet the clinical requirements.

The mNGS technology has been developing for several years (Inglis and Edwards, 2022). With the development of technology, the diagnostic efficiency of mNGS has been greatly improved, which has greatly enhanced the ability of clinicians to diagnose infectious diseases (Miao et al., 2018). Traditionally, auxiliary examination methods for the diagnosis of IPA include chest imaging findings, direct evidence of *Aspergillus* infection (such as microscopy or culture of biopsy, strong galactomannan positive in serum or bronchoalveolar lavage fluid, or *Aspergillus* polymerase chain reaction) (El-Baba et al., 2020). Chest imaging findings are often atypical, and it is frequent appearance that the same disease present with different images and the same image represent different diseases (Hage et al., 2019). Tissue biopsy procedure, an invasive examination method, can lead to great damage to the patient, especially in critically ill patients, causing a large proportion of patients’ tissue specimens cannot be obtained (Yu et al., 2020). Fungal culture of upper respiratory tract secretions lags clinical practice because of the long culture period and high false positive and false negative rates (Saubolle and McKellar, 2001). *Aspergillus* PCR is a confirmatory test that relies on existing nucleic acid sequences, with a narrow detection range (White et al., 2015). The mNGS technology, an exploratory detection method, is based on the sequencing and analysis of microorganisms and host nucleic acids in clinical samples, which can detect a variety of pathogenic microorganisms without bias. It can directly, rapidly, and objectively identify a large number of pathogenic microorganisms (viruses, bacteria, fungi, parasites) in clinical samples (Miao et al., 2018; Gu et al., 2019). However, due to its high sensitivity, it also brings certain clinical troubles, especially for opportunistic pathogens. When the number of mNGS sequence is relatively low, it is hard to determine whether the pathogens is pathogenic, such as *Aspergillus*. Therefore, our research used the histopathological results of admitted patients as the gold standard to evaluate the diagnostic efficacy of mNGS for IPA.

Our research indicated that mNGS in BALF for IPA had a good diagnostic efficacy in whole patient population when the cut-off value of SSRN of mNGS was 2.5. At this point, the Youden index was the largest, and the sensitivity, specificity and accuracy synthesis is optimal (AUC, 0.790; sensitivity, 73.6%; specificity, 83.2%; accuracy, 80.5%, respectively). However, when applying the SSRN of mNGS in different populations, the cut-off values should be adjusted accordingly (the cut-off values of mNGS sequences in immunosuppressed subgroup and diabetic subgroup were 1 and 4.5, respectively). In the immunocompromised population, when the cut-off value was adjusted from 2.5 to 1, the corresponding AUC increased from 0.742 to 0.788, the balance accuracy (sensitivity + specificity/2) increased from 74.15% to 78.75%, and the accuracy did not alter. While the ROC curve after adjusting the cutoff value by the Youden index showed that the balance accuracy was higher than the accuracy, several studies reported accuracy rather than balance accuracy (Subasi, 2012; Wei and Dunbrack, 2013), which could be more realistic indicator. In the diabetic population, when the cut-off value was adjusted from 2.5 to 4.5, the corresponding AUC increased from 0.793 to 0.832, and the accuracy increased from 79.4% to 85.3%. Moreover, our study found culture in sputum and BALF and GM test in blood and BALF had no statistically significant difference between Proven IPA and Proven non-IPA-PD groups (*P* > 0.05). This may be due to the low positive rate (9/176) and sensitivity (1/50) of sputum culture. Meanwhile, the positive rate and the sensitivity of BALF culture were also low (31/170 and 7/43, respectively). Because of additional fungal diseases in the Proven non-IPA-PD group, including mucormycosis, cryptococcosis, candidiasis, etc. (27/89), which contributed to an increased positive rate of GM test, there was no statistically significant difference in GM test in serum and BALF between the two groups (*P* > 0.05). In addition, the samples were almost all from non-neutropenic patients in our study and neutrophils can clear GM in serum (Koulenti et al., 2014), and this could also lead to no statistically significant difference in GM test in serum between two groups, which was consistent with previous studies (Vergidis et al., 2020; Arastehfar et al., 2021). Although studies on the diagnostic performance of mNGS for IPA were plentiful (Hoenigl et al., 2023), studies using histopathology as the gold standard are still lacking (Peng et al., 2021). To the best of our knowledge, this research is the first comparative study of the diagnostic efficacy of mNGS using histopathology as the gold standard.

Despite the rigorous design and careful analysis, our study has limitations. The diagnosis of invasive pulmonary aspergillosis based on histological microbial morphology is not sufficiently accurate. A definitive diagnosis still requires confirmation by positive *Aspergillus* culture or positive *Aspergillus* specific PCR test on biopsy tissue. Because of the tiny amount of tissue obtained by lung biopsy, it was difficult to obtain enough tissue for *Aspergillus* culture and PCR testing, but our proven IPA cases were mutually verified by two experienced independent pathologists according to the characteristic morphology of *Aspergillus*. The mNGS can detect many microorganisms, including parasitic bacteria, colonizing bacteria, background bacteria and pathogenic bacteria, but it cannot determine which is the pathogenic bacteria or which is the dominant pathogen, and there are no drug sensitivity results to guide clinical practice (Miao et al., 2018; Simner et al., 2018). Therefore, mNGS cannot replace traditional pathogen culture. Making clinical decisions, clinicians must accord to the specific clinical condition, which need to combine the patient’s immune status, symptoms, physical examination, comprehensive conventional microbiological tests, inflammatory, serological, imaging, and other indicators.

## 5 Conclusions

The mNGS in BALF has a high diagnostic efficacy for proven IPA, superioring to *Aspergillus* culture in sputum and BALF and GM test in blood and BALF. However, the cut-off value of SSRN should be adjusted when in different population.

## Data availability statement

The data presented in the study are deposited in the ENA repository(https://www.ebi.ac.uk/ena/browser/), accession number PRJEB63639.

## Ethics statement

The studies involving humans were approved by Scientific Research and Clinical Trial Ethics Committee of the First Affiliated Hospital of Zhengzhou University. The studies were conducted in accordance with the local legislation and institutional requirements. The participants provided their written informed consent to participate in this study.

## Author contributions

HJ and HL designed the study. YW collated the data. MT carried out data analyses. HJ produced the initial draft of the manuscript. XW and JL revised manuscript. GZ guided on the quality of the research. All authors have seen the manuscript and approved to submit to your journal, and there is no conflict of interest among the authors.
